# Pain in polycystic ovary syndrome: a comprehensive bedside to bench perspective on an underrecognized symptom

**DOI:** 10.3389/fphar.2025.1670766

**Published:** 2026-01-13

**Authors:** Lida Khodavirdilou, Jenny L. Wilkerson

**Affiliations:** Department of Pharmaceutical Sciences, Texas Tech University Health Sciences Center, Amarillo, TX, United States

**Keywords:** drug discovery, hyperandrogenism, insulin resistance, neuroinflammation, pain, PCOS, sex hormones

## Abstract

Polycystic ovary syndrome (PCOS) is a common endocrine disorder, with a reported worldwide prevalence of 5%–20% in women of reproductive age. It is defined primarily by hyperandrogenism, ovulatory dysfunction, and polycystic ovarian morphology. In addition to the reproductive and metabolic features of PCOS, patients often experience chronic pain, which is the most frequently reported symptom. Pain in the pelvic area, dysmenorrhea, bloating, and abdominal cramping are all common in PCOS patients. This paper reviews various factors that may influence pain in PCOS. Heightened inflammatory markers, such as interleukin-6, tumor necrosis factor alpha, and C-reactive protein may sensitize nociceptive pathways and promote an exaggerated immune response. Hyperinsulinemia and insulin resistance present in PCOS may further potentiate inflammatory processes. Alterations in sex hormones, specifically elevated androgens and a disproportionate estrogen to progesterone ratio, may alter nociceptive processing. Oxidative stress can potentiate sensitization through nociceptor hyperexcitability. We also discuss findings from animal models that mimic PCOS’s hormonal and behavioral aspects. Treatment modalities are reviewed, including hormonal therapies, insulin sensitizers, anti-androgens, lifestyle changes, as well as emerging therapies like agents that target the neuroimmune system and metabolic pathways. Overall, a deeper understanding of these interconnected mechanisms of this highly intertwined disorder is needed to help accurately diagnose it and improve the care of women experiencing PCOS-related pain.

## 1 Introduction

Polycystic ovary syndrome (PCOS) is a common endocrine disorder, affecting approximately 5%–20% of women of reproductive age worldwide (Riestenberg et al., 2022). The clinical markers for PCOS include hyperandrogenism, ovulatory dysfunction, as well as polycystic ovarian morphology, and diagnosis requires at least two of these factors under the Rotterdam criteria (Shukla et al., 2025; Nikanfar et al., 2024). PCOS causes metabolic, inflammatory, and psychological problems that significantly impact quality of life beyond reproductive issues (Shukla et al., 2025). Psychiatric conditions like depression, as well as anxiety, along with disordered eating habits, remain common, and these conditions can worsen both physical symptoms and treatment processes (Sacca et al., 2024). The multiple concurrent complications of PCOS result in significant healthcare costs and clinical burdens. It is estimated that over 5 million women in the United States suffer from PCOS, which leads to roughly $4 billion in healthcare costs every year (Sacca et al., 2024). Most research has focused on reproductive and metabolic features of PCOS, but emerging evidence suggests that pain, particularly chronic pain, may be an under-recognized component of the syndrome (Sacca et al., 2024). Patients with this condition frequently report pelvic pain, abdominal cramping, dysmenorrhea, as well as bloating due to oligomenorrhea or amenorrhea, and experience cramping even when menstruation is absent (Sacca et al., 2024; Lu et al., 2022; Cherlin et al., 2024). A 2017 qualitative study found that 27.6% of patient complaints in women with PCOS were related to pain and discomfort, and were overall the most frequently reported symptoms (Martin et al., 2017). Sex differences play a fundamental role in how pain is processed and maintained. Experimental and clinical research has shown that females and males often rely on distinct cellular and molecular pathways for nociceptive processing, influenced by hormonal and immune factors. Preclinical studies demonstrate that microglial activation within the spinal cord primarily mediates pain hypersensitivity in males, whereas females depend more on T cell–driven immune signaling to maintain neuropathic pain states (Mogil et al., 2024; Sorge et al., 2015). In visceral and inflammatory pain models, glial and neuroimmune interactions contribute disproportionately to persistent pain in females, suggesting a biological basis for the higher prevalence of chronic pain disorders among women (Dodds et al., 2016). Complementing these findings, Nicotra et al. (2014) developed a refined graded nerve injury and von Frey assessment approach to quantify sex differences in mechanical allodynia, revealing exaggerated and hormone-dependent tactile hypersensitivity in female rats across estrous phases (Nicotra et al., 2014). This study highlights both methodological and biological factors that shape female pain sensitivity and supports the concept that hormonal cycling directly influences nociceptive thresholds. Collectively, these findings underscore the importance of incorporating sex as a biological variable in pain research, particularly for female-specific conditions such as PCOS, where hormonal dysregulation and inflammation may further modify pain pathways.

Multiple biological mechanisms contribute to PCOS-related pain, including chronic low-grade inflammation and oxidative stress, along with adipogenesis and hormonal imbalances due to insulin resistance and hyperandrogenism (Sacca et al., 2024; Lu et al., 2022). These factors may sensitize pain pathways and disrupt neuroimmune signaling. Though the specific causal connections remain unclear, women with PCOS experience a higher likelihood of developing chronic pain syndromes such as fibromyalgia, migraines, and irritable bowel syndrome (Lu et al., 2022). As both exhibit pelvic pain and inflammatory pathophysiology, PCOS often clinically overlaps with endometriosis (Lu et al., 2022). Tools that measure health-related quality of life, including Polycystic Ovary Syndrome Health-Related Quality of Life and Short Form-36 (SF-36) consistently show reduced scores in the bodily pain domain among women with PCOS compared to healthy controls, emphasizing pain’s measurable impact on health-related quality of life (Cherlin et al., 2024; Li et al., 2011; Elsenbruch et al., 2003). Standard diagnostic criteria for PCOS do not include pain, and clinical care frequently ignores this symptom. Pain is often addressed as a secondary symptom, highlighting the need for more focused research and tailored management strategies (Lu et al., 2022).

Our review aims to combine existing evidence on pathophysiological mechanisms that connect PCOS with pain through hormonal, metabolic, inflammatory, and oxidative factors as well as evaluates its clinical manifestations and treatment methods alongside research models. The review underscores the need to include pain management in the ongoing clinical treatment of PCOS patients and suggests more research is needed to develop better individualized treatments that enhance patient results.

## 2 Impact on quality of life

PCOS impacts many aspects of quality of life, particularly mental health. This is critically important, as numerous studies have linked poor mental health to worse pain outcomes and pain catastrophizing (Woo, 2010; Scott et al., 2016; Quartana et al., 2009). Research indicates that nearly 60% of PCOS women experience moderate to severe psychological distress, with significantly higher risks of depression (1.65 times) and anxiety (1.42 times) compared to women without the condition (Yadav et al., 2023; Rowlands et al., 2016). Moreover, eating disorders (11.0% vs. 7.6%), low self-esteem (31.7% vs. 24.2%), and severe psychological distress (21.0% vs. 13.5%) are reported at higher rates by these women (Tay et al., 2019). Physical symptoms, like obesity (affecting 37% of women with PCOS) and irregular periods (55.4%), exacerbate these mental disorders and lead to social and occupational impairment (Rowlands et al., 2016). Qualitative reports suggest that some patients feel their concerns are not fully addressed during early clinical encounters, which may contribute to delays in recognizing symptom burden, including pain (University of Colorado Anschutz Medical Campus, 2025).

## 3 Types of pain associated with PCOS

Numerous types of pain are linked to PCOS, which may have a negative effect on a patient’s quality of life. Research has shown that women with PCOS may have altered pain processing systems, possibly impacted by chronic low-grade inflammation (Lu et al., 2022). Pelvic pain is particularly common among PCOS patients (Lu et al., 2022). While the exact mechanisms need further investigation, several physiological pathways could be involved, including alterations in the hypothalamic-pituitary axis and endogenous opioid system (Lonardo et al., 2024; Eyvazzadeh et al., 2009). Pelvic pain in this population is multifactorial and may arise from PCOS-related hormonal and inflammatory dysregulation, but comorbid endometriosis should also be carefully considered, as it can present with overlapping symptoms and shared mechanisms (Lu et al., 2022). Epidemiologic evidence indicates that true co-occurrence of PCOS and endometriosis is relatively uncommon in the general population (≈0.02%) (Jeong et al., 2024), yet higher rates are reported in clinical or surgical cohorts of women presenting with pelvic pain (≈5%) (Schliep et al., 2023). Notably, markedly higher frequencies, up to 70%, have been observed among women hospitalized for PCOS or those with self-reported infertility and/or pelvic pain (Holoch et al., 2014), reflecting selection bias in symptomatic populations. This suggests that while endometriosis is not universally responsible for pelvic pain in PCOS, it may account for a substantial proportion of pain cases in those seeking care. Furthermore, women diagnosed with either endometriosis or PCOS tend to report chronic pelvic pain more often than those without these conditions, but menstrual pain (dysmenorrhea) appears to be most pronounced when both disorders coexist (Schliep et al., 2023). Both disorders exhibit chronic low-grade inflammation, altered steroid hormone signaling, and neuroimmune sensitization, supporting the possibility of partially shared pathophysiologic pathways that may amplify pain even in the absence of overt endometriotic lesions (Orisaka et al., 2023).

Research conducted with the SF-36 questionnaire has found that PCOS patients scored much lower on the bodily pain scale than healthy controls. On the SF-36 scale (0–100, higher scores indicate less pain), Elsenbruch et al. found an average pain score of 73 in PCOS compared with 85 in controls, suggesting higher rates of pain in PCOS (Lu et al., 2022). Another PCOS-related symptom is cramping, a painful, long-term muscle contraction lasting from a few seconds to several minutes (Bordoni et al., 2024). It is most commonly experienced during menstrual periods, but in PCOS, it can occur outside of menstruation, as reported by 70% of patients in a study (Martin et al., 2017). Such cramps are often intense and unrelated to ovulation or menstruation, further exacerbating PCOS symptoms. Although this is a commonly expressed concern by patients, clinicians tend to overlook the significance of cramping in PCOS (Martin et al., 2017). Another common symptom reported by some individuals with PCOS is dysmenorrhea, or painful menstruation. While not a defining feature of PCOS, studies in young women have identified a strong correlation between polycystic ovarian morphology (PCOM) and the severity of primary dysmenorrhea (Jeong et al., 2019). PCOM, characterized by multiple small follicles on ovarian ultrasound, is one of the diagnostic criteria for PCOS (Rocha et al., 2019). Women with PCOM were more likely than those without PCOM to experience severe menstrual pain (Jeong et al., 2019). Several mechanisms may explain this association. Elevated levels of prostaglandin E2 (PGE2) and prostaglandin F2α have been reported in women with heavy menstrual bleeding and dysmenorrhea, two conditions commonly observed alongside PCOM (Jeong et al., 2019). These prostaglandins are known to cause myometrial and uterine blood vessel contractions, contributing to menstrual pain (Csapo, 1980; Xholli et al., 2021). Furthermore, endothelial dysfunction may also play a role, as suggested by elevated levels of asymmetric dimethylarginine, an inhibitor of nitric oxide synthase that may impair uterine blood flow (Huang et al., 2012). Together, these findings support a role for inflammatory and vascular factors in the increased menstrual pain experienced by women with PCOM (Jeong et al., 2019).

The endogenous opioid system, particularly β-endorphins, is important in pain modulation and reproductive function (Böttcher et al., 2017). Women with PCOS have been found to have elevated levels of plasma β-endorphin, which might contribute to altered pain perception and metabolic disruption (Eyvazzadeh et al., 2009). Another PCOS-related pain condition is dyspareunia or painful sexual intercourse. Meta-analysis found that women with PCOS had significantly lower scores on the pain subscale of the Female Sexual Function Index than women without PCOS (Pastoor et al., 2018; Loh et al., 2020). This shows that PCOS women tend to experience more discomfort during sexual activity (Pastoor et al., 2018; Loh et al., 2020). It is not yet known what drives PCOS-related dyspareunia, but it could be related to several factors. Hormonal dysfunction, such as excess androgen production and potential estrogen deficiency, can lead to vaginal atrophy, which may result in painful intercourse (Loh et al., 2020). Psychological factors such as depression and anxiety are significantly more prevalent among individuals with PCOS; reported depression rates range from 16% to 55.6% and may contribute to sexual dysfunction (Dybciak et al., 2023). Women with PCOS have a higher prevalence of female sexual dysfunction (35%) compared to those without PCOS (29.6%) (Loh et al., 2020). Mittelschmerz, or ovulation pain, is a midcycle abdominal discomfort experienced by over 40% of women of reproductive age (Brott and Le, 2023). The pain is mild to severe and is linked to the rapid expansion of the dominant follicle just before ovulation, rather than the follicular rupture itself (Poonam et al., 2013; Brott and Le, 2023). In women with PCOS, the experience of mittelschmerz may be less common due to the chronic anovulation and irregular ovulation patterns associated with the condition (Harris et al., 2017). The connection between PCOS and mittelschmerz requires further study.

Emerging evidence suggests that pain experiences in PCOS may differ across clinical phenotypes, reflecting variability in hormonal, inflammatory, and metabolic profiles. Women with phenotypes characterized by pronounced metabolic or inflammatory dysfunction, such as oligo/amenorrhea with polycystic ovarian morphology, tend to exhibit higher levels of systemic inflammatory markers, which are known contributors to pain sensitivity (Ozkan et al., 2025). In contrast, hyperandrogenic phenotypes have been associated with altered adiposity, neuroendocrine activity, and nociceptive processing (Dumesic et al., 2016; Eichwald and Talbot, 2020). Despite these observations, no large-scale studies have yet compared the prevalence or severity of chronic pain among distinct PCOS subtypes, underscoring the need for future research to clarify whether specific phenotypic profiles confer heightened pain vulnerability (Sacca et al., 2024).

## 4 Theories on the pathophysiology of pain in PCOS

### 4.1 Hormonal imbalances

**FIGURE 1:**
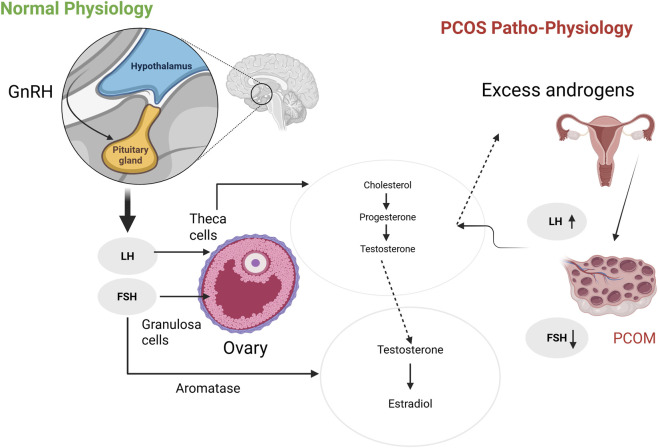
Schematic representation of normal ovarian physiology and pathophysiological mechanisms leading to hyperandrogenism in Polycystic Ovary Syndrome. In the normal reproductive axis (left), gonadotropin-releasing hormone (GnRH) from the hypothalamus stimulates the pituitary to release luteinizing hormone (LH) and follicle-stimulating hormone (FSH). LH stimulates theca cells in the ovary to produce androgens, which are then converted to estradiol by granulosa cells under FSH stimulation via aromatase activity. In polycystic ovary syndrome (PCOS; right), an increased LH/FSH ratio leads to excessive androgen production by theca cells and insufficient FSH stimulation of granulosa cells. This hormonal imbalance reduces androgens conversion to estrogens, follicular arrest, and the development of polycystic ovarian morphology (PCOM). This imbalance contributes to anovulation and reproductive dysfunction. Created in BioRender.

Hormonal dysregulation forms the basis of PCOS through excess androgen production and disrupted estrogen and progesterone balance. In physiological conditions, pulsatile gonadotropin-releasing hormone (GnRH) regulates luteinizing hormone (LH) and follicle-stimulating hormone (FSH) (Filicori, 2023). LH primarily acts on theca cells to produce androgens, while FSH stimulates aromatase activity in granulosa cells to convert androgens into estradiol, which supports follicular maturation (Latronico and Segaloff, 1999). Figure 1 illustrates normal ovarian physiology and its disruption in PCOS. In PCOS, chronic anovulation leads to consistently low progesterone levels, contributing to an elevated LH-to-FSH ratio (Tsutsumi and Webster, 2009; Casteel and Singh, 2025; Emanuel et al., 2022). Increased LH further enhances ovarian androgen production, while reduced FSH impairs aromatase activity, reinforcing hyperandrogenism and endocrine dysregulation (Latronico and Segaloff, 1999; Emanuel et al., 2022; Hussein et al., 2024). Hyperandrogenism is a key clinical hallmark of PCOS and shapes the hormonal environment in which pain is experienced (Evans et al., 2021a). The hormonal profile characteristic of PCOS, low progesterone, altered estrogen signaling, and hyperandrogenism may influence nociceptive processing and contribute to pain susceptibility in affected individuals (Lu et al., 2022).

Hormonal dysregulation is increasingly linked to both acute and chronic pain disorders, with sex hormones, such as estrogen, progesterone, and testosterone being important pain modulators (Blackburn-Munro and Blackburn-Munro, 2003). These effects have been documented across a range of conditions including rheumatoid arthritis, irritable bowel syndrome, chronic pelvic pain, fibromyalgia, and temporomandibular joint disorder, all of which are more prevalent in women (Blackburn-Munro and Blackburn-Munro, 2003; Averitt et al., 2019). Pain sensitivity in women fluctuates across the menstrual cycle, often peaking during phases of high estrogen (Athnaiel et al., 2023). For example, Bajaj et al. found that women had the highest sensitivity to heat pain during the ovulatory phase compared to menstrual, follicular, and luteal phases, with significantly lowered thresholds (Bajaj et al., 2001). While progesterone and testosterone are generally associated with analgesic effects, the influence of estrogen is complex and context-dependent, with evidence showing both pro-nociceptive and anti-nociceptive actions (Schertzinger et al., 2018). Across experimental and clinical studies, higher estrogen states are often associated with lower pain thresholds and greater sensitivity in both somatic and visceral modalities (Chaban, 2013; Amandusson and Blomqvist, 2013), whereas progesterone is more consistently linked to dampening of pain responses, likely through enhancement of inhibitory tone and reduction of stress- and inflammation-related influences on pain (Athnaiel et al., 2023; Hornung et al., 2020). Testosterone has also been associated with reduced experimental pain sensitivity and engagement of endogenous analgesic systems (Wu-Chen et al., 2025). Taken together, these findings suggest that the balance between estrogen-driven facilitation and progesterone- and testosterone-supported inhibition is a critical determinant of overall pain sensitivity; in PCOS, chronic low progesterone combined with altered estrogen dynamics and hyperandrogenism may therefore shift this balance toward a hormonal milieu that favors heightened pain sensitivity and greater vulnerability to pelvic and visceral pain (Lu et al., 2022).

In a rat model of nerve injury, ovary-intact females were more susceptible to mechanical allodynia, or light touch mechanical sensitivity, than males or ovariectomized females, indicating an effect of endogenous estrogens on pain sensitivity (Coyle et al., 1996). Studies involving combination oral contraceptive pill users with low endogenous estradiol and testosterone levels have shown that the low estrogen environment, which is comparable to the menstrual cycle’s early follicular phase, might result in enhanced pain sensitivity (Vincent et al., 2013). PCOS can aggravate conditions like dysmenorrhea, endometriosis, and irritable bowel syndrome, conditions linked to pelvic pain and sexual dysfunction due to underlying hormonal imbalance (Lu et al., 2022). Furthermore, ovarian hormones frequently regulate chronic non-cancer pain problems, which are more common in women. This suggests that hormonal fluctuations associated with PCOS may exacerbate these conditions during the prime years of reproduction (Hassan et al., 2014). Despite the influences of hormonal dysregulation on pain in PCOS, the underlying mechanisms are not well understood.

### 4.2 Insulin resistance and its role in pain

Insulin hormone is essential to maintain glucose levels. It is linked to insulin resistance, a condition in which the body’s cells become less responsive to the effects of insulin, resulting in high insulin and harmful hyperinsulinemia. This can lead to various metabolic disorders and related diseases, such as type 2 diabetes and obesity (Szablewski, 2024). Within PCOS insulin resistance is a powerful pathophysiological link in the development of the syndrome and is observed in a considerable number of PCOS patients (Xu and Qiao, 2022). The correlations between insulin resistance and PCOS are complicated and bidirectional. Enhanced LH secretion and direct interaction with ovarian theca cells may increase ovarian androgen production due to chronic hyperinsulinemia from insulin resistance (Unluhizarci et al., 2021). Furthermore, hyperinsulinemia is known to increase the bioavailability of androgens such as testosterone through the reduction of the sex hormone-binding globulin (SHBG) (Riayati et al., 2023).

Androgens themselves can exacerbate insulin resistance by impairing insulin signaling pathways, creating a vicious cycle that perpetuates metabolic and endocrine PCOS abnormalities (Unluhizarci et al., 2021). Insulin resistance can contribute to pain through several mechanisms. Insulin resistance is sufficient to stimulate chronic low-grade inflammation, which is essential in PCOS and PCOS-related pain (Orisaka et al., 2023). High glucose levels can also induce oxidative stress, known to contribute to pain sensitization (Ye et al., 2022; Doyle and Salvemini, 2021; Shim et al., 2019), as well as proinflammatory actions in mononuclear cells, enhancing adipocyte cytokine production (Purwar and Nagpure, 2022). The role of specific proinflammatory cytokines, second messenger signaling molecules as well as oxidative stress in PCOS-related pain is discussed in greater detail below. It has been reported that women with PCOS have higher pain perception compared to healthy controls; this is partly attributed to obesity and infertility, both of which are enhanced by insulin resistance (Lu et al., 2022). The deregulation of inflammatory cytokines, adipokines (cell signaling proteins secreted by adipose tissue) and insulin resistance are considered the most critical pathophysiological factors that contribute to the development or worsening of pain among PCOS patients (Elsenbruch et al., 2003). Hyperinsulinemia associated with insulin resistance is regarded as one of the main factors that impair oocyte quality, ovulatory function and sub-fertility (Hassan et al., 2023). These can lead to pelvic pain and sex difficulties in women with PCOS. The chronic pain experienced by PCOS patients is often overlooked in clinical settings despite evidence suggesting that pain perception is significantly higher in these patients compared to healthy controls (Lu et al., 2022).

### 4.3 Inflammatory markers and their contribution to pain

Recent research suggests that low-grade chronic inflammation underlies the pathogenesis of PCOS (Aboeldalyl et al., 2021). Women with PCOS have significantly increased levels of inflammatory markers, namely, cytokines and chemokines like interleukin-6 (IL-6), tumor necrosis factor-alpha (TNF-α), interleukin-17 (IL-17), and C-reactive protein (CRP), signifying a chronic inflammatory state (Kaur et al., 2024; Dey et al., 2023; Vasyukova et al., 2023; Özay and Özay, 2021). The importance of these cytokines and inflammatory proteins related to pain will be discussed below. These inflammatory cytokines and second messenger signaling molecules each contributes to metabolic and reproductive disturbances, including insulin resistance and obesity (Özdemir Başer et al., 2022; Abraham Gnanadass et al., 2021). This cycle is related to PCOS pathophysiology; it modifies ovarian function and increases the risk of developing metabolic and cardiovascular diseases (Abraham Gnanadass et al., 2021; Rudnicka et al., 2021). While obesity in PCOS is known to correlate with chronic low-grade inflammation due to enlarged adipocytes and immune cell infiltration within adipose tissue, elevated circulating levels of CRP have also been shown to be present in lean women with PCOS, pointing to inflammation as a significant aspect of the condition irrespective of obesity (Dey et al., 2023; Vasyukova et al., 2023; Escobar-Morreale et al., 2011). Furthermore, PCOS is associated with proinflammatory genotypes, including those linked to TNF-α, its cognate receptor, Tumor Necrosis Factor Receptor II, and IL-6, demonstrating a genetic predisposition for chronic low-grade inflammation (Dey et al., 2023; González, 2012). Increased inflammation markers in first-degree relatives of PCOS patients, like CRP and IL-6, further suggest a potential familial or genetic predisposition for inflammation and related metabolic concerns (Kaur et al., 2024; Pradhan et al., 2022a; Pradhan et al., 2022b). This evidence supports a hereditary role in the inflammatory processes in PCOS, independent of obesity. Although the relationship between inflammation and clinically reported pain in PCOS has not yet been characterized as extensively as in conditions like osteoarthritis (Slade et al., 2023), the available data strongly suggest similar inflammatory mechanisms. The underlying chronic inflammatory state is therefore expected to exacerbate pain through mechanisms similar to those described in other inflammatory disorders. In PCOS, elevated inflammatory markers are well positioned to contribute to increased pain sensitivity and to the development or maintenance of chronic pain conditions.

One of the most important inflammatory mediators in pain processing is IL-6. IL-6 is accepted as contributing to pain and hypersensitivity during inflammatory, neuropathic, and cancer pain by activating peripheral immune cells, central nervous system glia, and neurons along critical pain processing pathways (Kummer et al., 2021). IL-6 can sensitize nociceptors directly, leading to mechanical allodynia (pain from typically non-painful stimuli) and thermal hyperalgesia (increased sensitivity to pain from heat or cold) (Hisaoka-Nakashima et al., 2022; Sebba, 2021), while an intrathecal injection of anti-IL-6 neutralizing antibody can significantly reduce these pain-related behaviors (Zhou et al., 2016a). Evidence for this comes from a study by Marino et al., which found that modulation of the IL-6 pathway attenuated mechanical hyperalgesia and thermal hypersensitivity in a reserpine-induced rat model of fibromyalgia (Marino et al., 2023). As an important component of pain processing, targeting interleukin-6 could be a promising lead to novel therapeutic strategies for pathological pain (Zhou et al., 2016a).

TNF-α is another inflammatory mediator that may contribute to pain sensitivity in PCOS (Lu et al., 2022). This cytokine is involved in many types of pain, from acute inflammation to chronic neuropathic pain (Sorkin and Doom, 2000; Zhang and An, 2007). TNF-α triggers what is commonly known as a cytokine storm and amplifies a cascade of pain-related pro-inflammatory cytokines; it also induces and modulates neuropathic pain by enhancing sensitization at the level of the periphery via acting at first-order afferents and also centrally via spinal cord nociceptors (Duan et al., 2022). TNF-α-induced analgesia has been demonstrated by using different experimental models. For instance, Sorkin and Doom et al. showed that applying TNF-α to the epineurium of the sciatic nerve causes acute mechanical hyperalgesia (Sorkin and Doom, 2000). Zelenka et al. also demonstrated that physiological doses of TNF-α injected into the sciatic nerve of rats caused pain behavior without nerve damage (Zelenka et al., 2005).

IL-17, a well characterized pro-inflammatory cytokine, has been identified to play critical roles in pain processing and chronic inflammatory pain development (Luo et al., 2019a). IL-17 contributes to pain hypersensitivity by indirect and direct mechanisms (Jiang et al., 2022). Indirectly, IL-17 regulates immune cell infiltration and stimulates the production of pain mediators, particularly TNF-α (Jiang et al., 2022). Since IL-17 interacts with TNF-α, studies have shown that hyperalgesia caused by IL-17 might depend on TNF-α binding to Tumor Necrosis Factor Receptor 1 (TNFR1) to trigger neutrophil migration and the production of analgesic mediators, resulting in increased nerve sensitivity (McNamee et al., 2011). In the study by Richter et al., in rats intraarticular injection of IL-17A directly activates nociceptors, leading to a slow-developing but long-lasting sensitization of nociceptive C fibers and mechanical hyperalgesia development (Richter et al., 2012). IL-17 has also been found to be a critical pain mediator in chronic pelvic pain conditions, including endometriosis and prostatitis (Shi et al., 2022; Liu et al., 2017). In studies by Liu et al. and Murphy et al. utilizing rodent models of experimental autoimmune prostatitis, IL-17 was upregulated and essential for developing pelvic pain (Liu et al., 2017; Murphy et al., 2015). These findings, along with the known involvement of IL-17 in endometriosis-related pain, suggest that IL-17 could be a common factor in various pelvic pain conditions. Given that IL-17 is a critical mediator in several pelvic pain conditions and that a subset of women with PCOS report pelvic pain (Lu et al., 2022), it is plausible that IL-17 contributes to pain generation in PCOS as well, making IL-17 signaling a biologically credible therapeutic target that warrants direct investigation in this population. Furthermore, IL-17 signaling blockade has shown promise in reducing pain in various inflammatory models. Intraarticular injection of IL-17 caused joint mechanical hypernociception in a mouse model of antigen-induced arthritis, and this was reversed by IL-17 neutralization (Pinto et al., 2010). In a mouse model of experimental autoimmune prostatitis, prophylactic anti-IL-17 treatment prevented pelvic mechanical allodynia from developing but failed to reverse existing pain (Murphy et al., 2015).

C-reactive protein, an acute-phase protein synthesized by the liver and by several extrahepatic sources such as smooth muscle cells, macrophages, lymphocytes, and adipocytes, has been linked to inflammatory pain hypersensitivity and is a causative factor in pain processing (Hsuchou et al., 2012; Pepys and Hirschfield, 2003; Mouliou, 2023). CRP caused mechanical and thermal hyperalgesia in rats after intraplantar injection, and overexpression in the dorsal root ganglion (DRG) correlated with increased pain behaviors, indicating a potential role for CRP at the DRG level in modulating nociceptive signaling (Liu et al., 2023). Moreover, CRP activates complement cascades and induces expression of proinflammatory cytokines, such as interleukin-6 and TNF-α (Friend et al., 2022; Zhou et al., 2016b; Sproston and Ashworth, 2018), and through this mechanism, is indirectly involved in pain sensitization (Zhang and An, 2007). Similarly, via the leaky blood-brain barrier during inflammation (Galea, 2021), CRP enters the central nervous system, where it stimulates microglia and astrocytes to activate central sensitization (Hsuchou et al., 2012). Elevated CRP was associated with higher prevalence of pain in older adults, even after adjustment for other factors, as reported by Sturmer et al. (2005). Furthermore, Afari et al. showed that CRP levels were positively linked to pain intensity in patients with chronic widespread pain (Afari et al., 2011). In the context of PCOS, women diagnosed with PCOS have been found to have higher CRP levels compared to healthy controls (Escobar-Morreale et al., 2011). This systematic chronic low-grade inflammation is therefore likely to contribute to pain symptoms experienced by some women with PCOS, particularly pelvic pain, via mechanisms involving peripheral and central sensitization (Lu et al., 2022). However, further research is needed to understand CRP’s contribution and potential as a target for treating PCOS-related pain. Overall, the convergence of elevated IL-6, TNF-α, IL-17, and CRP levels in PCOS with their well-established roles in sensitizing peripheral nociceptors, activating spinal and supraspinal glia, and promoting central sensitization supports a model in which chronic low-grade inflammation is a major driver of pain in PCOS. In this framework, inflammatory signaling interacts with hyperandrogenism, insulin resistance, and structural changes in reproductive tissues to lower pain thresholds and facilitate the development of pelvic pain and comorbid chronic pain syndromes. Future studies that simultaneously quantify inflammatory mediators and validated pain outcomes in women with PCOS will be essential to test this inflammatory pain model and to identify immune-related therapeutic targets.

### 4.4 Neuroimmune and endometrial pathways

Recent work highlights innate immune activation within the endometrium as a key contributor to chronic pelvic pain. In women with endometriosis, Toll-like receptor 4 (TLR4) is upregulated in macrophages and endometrial stromal cells, where stimulation with heat-shock protein 70 or lipopolysaccharide (LPS) enhances secretion of proinflammatory cytokines such as IL-6 and TNF-α, and promotes stromal cell proliferation effects that can be blocked by TLR4 inhibition (Khan et al., 2008). This indicates that innate immune receptors in the uterine microenvironment actively participate in pain-relevant inflammatory signaling. Under physiological conditions, menstruation represents a tightly controlled, hormone-driven inflammatory event (Salamonsen and Woolley, 1999). The fall in progesterone during the late luteal phase initiates a controlled inflammatory response within the endometrium, characterized by the influx of leukocytes, production of cytokines and prostaglandins, and activation of matrix metalloproteinases that mediate tissue breakdown and subsequent repair (Maybin and Critchley, 2015). This transient inflammatory phase normally resolves as progesterone levels rise again in the next cycle. However, when progesterone signaling is deficient or functionally resisted, as reported in both PCOS and endometriosis, the resolution phase is disrupted, resulting in persistent cytokine production, prolonged leukocyte activation, and impaired endometrial homeostasis (Salamonsen and Woolley, 1999). Such dysregulation provides a mechanistic link between hormonal imbalance and chronic local immune activation, contributing to the sustained inflammatory milieu underlying pelvic pain in both conditions. Supporting this model, peripheral blood mononuclear cells from women with chronic pelvic pain release significantly greater amounts of IL-1β following lipopolysaccharide-induced TLR4 activation, compared with pain-free controls, indicating a state of peripheral immune hyper-responsiveness and systemic neuroimmune sensitization (Evans et al., 2020; Evans et al., 2021b). Together, these findings suggest that reduced progesterone responsiveness may allow sustained TLR4-mediated immune activation within the endometrium, generating chronic inflammatory signaling that interacts with nociceptive and autonomic circuits. Although PCOS and endometriosis arise through distinct initiating mechanisms, metabolic-hormonal dysregulation *versus* ectopic tissue implantation (Tsamantioti and Mahdy, 2025), both disorders exhibit abnormal endometrial progesterone signaling and chronic inflammatory signaling that may converge on a shared neuroimmune axis driving pelvic pain (Li et al., 2014; Chang et al., 2025). Supporting the involvement of TLR4 signaling in PCOS pathophysiology, recent preclinical work by Dominique Wiggins et al. (2023) demonstrated that genetic deletion of TLR4 in a letrozole-induced mouse model of PCOS markedly attenuated both reproductive and metabolic abnormalities (Dominique Wiggins et al., 2023). TLR4-knockout mice maintained regular estrous cycling, exhibited normalized luteinizing hormone and follicle-stimulating hormone levels, and showed reduced weight gain and improved glucose tolerance compared with wild-type controls (Dominique Wiggins et al., 2023). These findings indicate that TLR4-mediated inflammation contributes causally to the endocrine and metabolic abnormalities of PCOS, reinforcing the link between immune activation and hormonal dysregulation. Together with prior evidence from endometriosis, this supports a shared inflammatory–neuroendocrine pathway and highlights the need for comparative research on TLR4 signaling across gynecologic pain disorders.

### 4.5 Oxidative stress and pain sensitization

**FIGURE 2:**
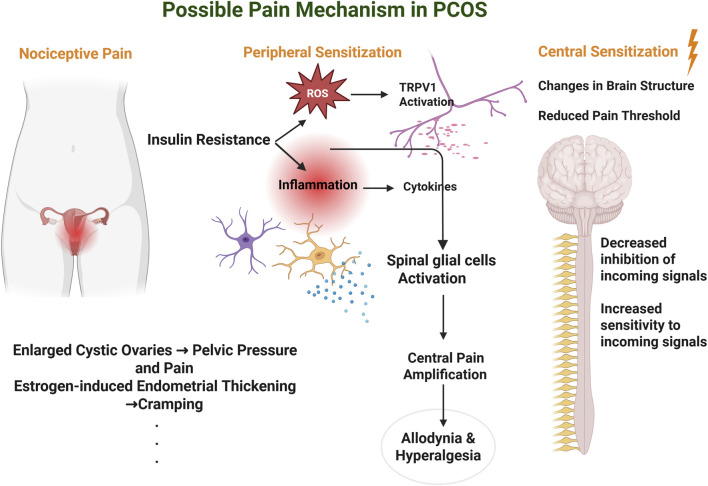
Possible Pain Mechanisms in Polycystic Ovary Syndrome. This figure shows the hypothesized pathways contributing to pain in PCOS, involving nociceptive, peripheral sensitization, and central sensitization mechanisms. Nociceptive pain arises from mechanical and inflammatory stimuli, such as enlarged cystic ovaries causing pelvic pressure and estrogen-induced endometrial thickening leading to menstrual cramping. Peripheral sensitization is driven by insulin resistance, which promotes chronic low-grade inflammation and reactive oxygen species (ROS) generation. ROS activates TRPV1 ion channels, while inflammatory cytokines sensitize peripheral nerves, lowering their activation threshold and amplifying pain perception. Central sensitization is triggered by sustained peripheral input and the spread of inflammatory signals to the spinal cord, where spinal glial cells become activated. This leads to central pain amplification, accompanied by brain structure changes and a lowered pain threshold, ultimately resulting in allodynia (pain from non-painful stimuli) and hyperalgesia (exaggerated pain response). Created in BioRender.

There is growing evidence that oxidative stress may be involved in the multifactorial pathophysiology of PCOS, as it can synergize with heightened levels of basal proinflammatory signaling molecules to worsen the disease (Zeber-Lubecka et al., 2023). Oxidative stress is a physiological condition initiated by an imbalance between the formation of free radicals, especially reactive oxygen species (ROS), and the body’s capacity to neutralize them through antioxidant mechanisms (Burton and Jauniaux, 2011; Correia and Vale, 2024). This balance is vital for preserving normal cellular activities, as antioxidants neutralize reactive species formed during metabolic reactions (Correia and Vale, 2024). The body’s antioxidant defense includes enzymatic systems such as superoxide dismutase, catalase, and glutathione peroxidase, which detoxify ROS and maintain redox homeostasis (Yamamoto et al., 2022). Studies have shown elevated oxidative stress markers in PCOS patients, including malondialdehyde, nitric oxide (NO), xanthine oxidase, and advanced glycation end products in the serum and erythrocytes of women with PCOS, along with a concurrent reduction in antioxidant defenses including superoxide dismutase, glutathione peroxidase, and total antioxidant capacity in serum, erythrocytes, and follicular fluid (Zuo et al., 2016; Artimani et al., 2018; Mohammadi, 2019). The oxidative imbalance in PCOS is linked with insulin resistance, hyperandrogenism and chronic low-grade inflammation, which are the central characteristics of the syndrome (Zuo et al., 2016). This oxidative stress is well connected to mitochondrial dysfunction in PCOS, which can cause cell energy deficits (Zeber-Lubecka et al., 2023). In various conditions with mitochondrial impairment, such cellular energy deficits have been associated with symptoms like fatigue and pain (Sui et al., 2013; Filler et al., 2014). Regarding pain more broadly, oxidative stress has been shown to be responsible for several chronic pain conditions. According to Bruehl et al., oxidative stress correlates with hallmarks of the pathological chronic pain phenotype (Bruehl et al., 2022). Oxidative stress can induce pain through a number of different pathways, including direct damage to cellular components, activation of inflammatory pathways, and sensitization of nociceptors (Bruehl et al., 2022). In particular, reactive oxygen and nitrogen species, including NO generated by inducible nitric oxide synthase, can activate transient receptor potential (TRP) channels, especially TRP ankyrin 1 (TRPA1) and TRP vanilloid 1 (TRPV1), which are key mediators of nociceptive signaling under oxidative and inflammatory conditions (Piciu et al., 2023). Higher levels of ROS can trigger peripheral and central nervous system hyperexcitability that can lead to hyperalgesia, even without nerve damage or tissue inflammation (Hendrix et al., 2020). Though inflammatory and oxidative mechanisms play roles in acute pain, ROS production seems particularly important in chronic pain (Hendrix et al., 2020). These hypothesized pathways are further detailed in Figure 2. Salvemini et al. showed that oxidative stress influences pain signaling on numerous levels of the nervous system (Salvemini et al., 2011). According to their study, superoxide and peroxynitrite contribute to inflammatory and neuropathic pain by central sensitization and increased peripheral sensitivity (Salvemini et al., 2011). Moreover, oxidative stress activates glial cells in the spinal cord, particularly microglia and astrocytes, which release proinflammatory cytokines and amplify nociceptive signaling (Zhu et al., 2022; Grace et al., 2016). PCOS is related to chronic low-grade inflammation and insulin resistance, which are associated with greater oxidative stress (González, 2012; Artimani et al., 2018). Although it has been understudied, the relationship between PCOS and pain is likely to include oxidative stress mechanisms and collectively represents an area where future research efforts are needed. Although hyperalgesia and prolongation of chronic pain have been linked to chronic systemic inflammation, signifying that a proinflammatory state may enhance pain perception in PCOS patients (Lu et al., 2022; DeVon et al., 2014; Watkins et al., 2003; Oliveira et al., 2014), more research is needed to elucidate whether and how inflammation might be involved in the pathogenesis of PCOS-related pain. Additional research should focus on the role of specific inflammatory markers in the emergence of pain in PCOS.

### 4.6 Structural abnormalities in reproductive organs

**FIGURE 3:**
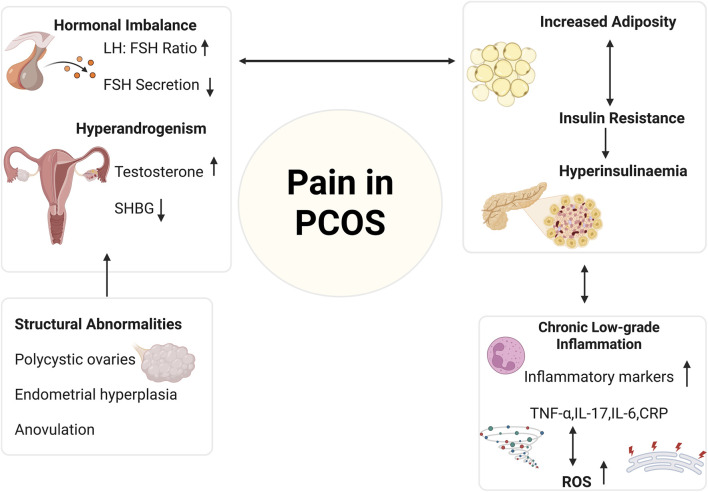
Multifactorial Mechanisms Contributing to Pain in Polycystic Ovary Syndrome. This figure shows the multifactorial biological contributors to pain in PCOS, highlighting the interconnected roles of hormonal, metabolic, inflammatory and structural factors. Hormonal imbalance is a key feature of PCOS, characterized by an increased LH: FSH ratio and decreased FSH secretion, leading to hyperandrogenism (elevated testosterone and reduced SHBG levels). These changes impair ovarian function and contribute to structural abnormalities, including polycystic ovaries, endometrial hyperplasia, and chronic anovulation, factors that can generate nociceptive pelvic pain. Metabolic dysfunction, such as increased adiposity and insulin resistance, leads to hyperinsulinemia, which promotes androgen excess and further exacerbates inflammation and structural changes. Chronic low-grade inflammation, marked by elevated cytokines (e.g., TNF-α, IL-6, IL-17) and ROS, sensitizes nociceptors and amplifies peripheral and central pain mechanisms. This model may best represent the complex nature of pain in PCOS. Created in BioRender. https://BioRender.com/x28y9ix.

PCOS is characterized by structural abnormalities in the female reproductive organs, including the formation of ovarian cysts and abnormal endometrial thickening (Xue et al., 2021). These structural features are linked to various clinical symptoms and possible long-term important health risks (Xue et al., 2021). However, current evidence suggests that pelvic pain in PCOS is rarely caused by ovarian follicles themselves but instead arises primarily from secondary pelvic processes that interact with the hormonal and metabolic environment characteristic of the syndrome (Saei et al., 2020). Pelvic floor muscle hypertonicity is highly prevalent among women with chronic pelvic pain, with high-tone pelvic floor disorder reported in 60%–90% of affected patients, and myofascial trigger points in the pelvic floor muscles are a recognized source of deep dyspareunia and sexual dysfunction (Torosis et al., 2024; Ross et al., 2021; Sandrieser et al., 2024). Although direct data in PCOS are limited, emerging work on pelvic floor dysfunction in PCOS and pelvic health clinical reports suggest that some women with PCOS may be at increased risk for pelvic floor muscle tightness and dyspareunia, potentially mediated by altered biomechanics, chronic abdominal or pelvic discomfort, and hormonal and metabolic factors (Saei et al., 2020; Kamal et al., 2025). Dysregulated uterine contractility, potentially influenced by low progesterone, altered estrogen signaling, and chronic anovulation, may also contribute to cramping-type pelvic pain and heightened sensitivity during menstrual or anovulatory cycles (MacLean and Hayashi, 2022; Itani et al., 2022). Cross-organ sensitization, a well-documented mechanism in chronic pelvic and visceral pain disorders, may further explain why women with PCOS frequently report overlapping symptoms such as bladder pain, bowel discomfort, and generalized pelvic hypersensitivity, as nociceptive input from reproductive organs can sensitize shared spinal and pelvic afferent pathways (Majima et al., 2021; Tseng et al., 2021; Malykhina, 2007). Together, these mechanisms likely contribute more to pelvic pain in PCOS than the structural ovarian features themselves. The interconnected nature of these multifactorial biological contributors to pain in PCOS is visually represented in Figure 3.

## 5 Clinical studies on pain management in PCOS

PCOS pain management clinical trials have included randomized controlled trials (RCT), observational trials, retrospective analyses and qualitative interviews. Retrospective studies, such as those using electronic health records from the TriNetX Global Network, have looked at the prevalence of pain in women with PCOS. According to this retrospective study, 19.21% of the 76.9 million women with PCOS reported having pain, with the largest prevalences seen in the Black or African American (32.11%) and White (30.75%) groups (Cherlin et al., 2024). Case-control studies have further examined menstrual pain, identifying an increased frequency of dysmenorrhea in women with PCOS, although pain intensity was found to be comparable to non-PCOS populations (Behzadfar et al., 2024). This pattern suggests that while the severity of each pain episode may not differ, women with PCOS experience pain more frequently or persistently, likely reflecting greater cycle irregularity, hormonal dysregulation, or altered nociceptive sensitivity. Thus, the overall burden of pain in PCOS remains clinically significant despite similar reported intensity levels.

RCTs have highlighted both the potential and limitations of various interventions. For example, an RCT on moxonidine in the treatment of musculoskeletal pain showed no pain reduction compared with a placebo, illustrating the difficulties of managing pain via sympathetic modulation (Jewson et al., 2020). An RCT of acupuncture in conjunction with herbal medicine, by contrast, found better reproductive outcomes, potentially impacting pain (Pan et al., 2022). A lifestyle intervention trial also demonstrated improvements in PCOS characteristics and phenotype severity (Dietz de Loos et al., 2021). While the study did not directly measure pain, these changes may contribute to symptom alleviation, including potential pain reduction. Qualitative studies have given a patient-centric perspective to pain management, which has highlighted how clinical specialists often underestimate pain and discomfort symptoms patients experience, including cramping, bloating and heavy bleeding, which have a profound effect on their quality of life (Martin et al., 2017). This disparity in clinical assessments and patient experiences highlights why addressing pain in all aspects of PCOS is important. Systems reviews and meta-analyses synthesized data from numerous studies to assess the effectiveness of interventions. For instance, a systematic review of lifestyle interventions for PCOS adolescents identified major changes in both anthropometric and clinical parameters. Although such changes are not directly related to pain, they can affect underlying processes, such as inflammation and obesity, which cause pain signs (Abdolahian et al., 2020).

## 6 Animal models of PCOS and preclinical pain research

Various animal models are used to replicate key features of PCOS, including hyperandrogenism, disrupted estrous cycles, metabolic dysfunction, and polycystic ovarian morphology. Hormonal models using aromatase inhibitors (e.g., letrozole) or non-aromatizable androgens (e.g., dihydrotestosterone, dehydroepiandrosterone), as well as combination models that pair these manipulations with high-fat diets, successfully reproduce many endocrine and metabolic characteristics of the human syndrome (Ul Haq Shah et al., 2022; Kafali et al., 2004; Krishnan et al., 2020; Morales-Ledesma et al., 2017; Begum et al., 2022; Roberts et al., 2017). Despite their widespread use, these models have rarely incorporated systematic assessment of pain-related behaviors, and no studies to date have specifically validated a preclinical PCOS model for chronic pelvic or widespread pain. By contrast, rodent endometriosis models and other gynecologic pain paradigms routinely include standardized measures of mechanical and thermal hypersensitivity, alongside assays that capture affective and functional consequences of pain (Tejada et al., 2023; Dodds et al., 2025). Adapting these behavioral approaches to existing PCOS models, while accounting for species differences in reproduction and for sex-specific considerations in preclinical pain research, represents an important future direction for elucidating endocrine–immune contributions to pain in PCOS.

## 7 Pharmacological and non-pharmacological approaches for PCOS symptom management

Hormonal treatments are at the core of treating PCOS-associated symptoms. Oral contraceptive pills (OCPs) serve as first-line treatments for menstrual irregularities, acne and hirsutism (Oguz and Yildiz, 2021; Shah et al., 2018). Among these, combined oral contraceptives (COCPs) containing both estrogen and progestin provide added benefits, including alleviating dysmenorrhea, menorrhagia, and endometriosis-related pain, while reducing the risk of endometrial, ovarian, and colon cancers (Dokras, 2016). COCPs work by suppressing LH and ovarian androgen production, increasing sex hormone-binding globulin, and reducing free testosterone (Shah et al., 2018; Georgia, 2024). Formulations with anti-androgenic progestins, like drospirenone or cyproterone acetate, are particularly effective in addressing hyperandrogenic symptoms (Oguz and Yildiz, 2021). Compared to conventional cyclic regimens, continuous or extended-cycle COCP regimens offer more androgen suppression and reliable symptom alleviation (Dokras, 2016). Progestin-only therapies also work for women with contraindications to estrogen-containing formulations, particularly in managing endometrial health (Shah et al., 2018). In addition to hormonal treatments, insulin sensitizers like metformin target metabolic dysregulation. While less effective than COCPs for menstrual cycle regulation, metformin improves insulin resistance and inflammation, particularly in patients with a BMI >25 kg/m^2^ (Georgia, 2024; Dunaif, 2008; Dhindsa et al., 2004). Medications like liraglutide and semaglutide, which are used for weight loss in obese patients, further support metabolic improvements, indirectly alleviating PCOS severity (Cena et al., 2020; Keating and Wild, 2023). Since 19.21% of women with PCOS have substantial pain symptoms, effective pain management should be an essential part of PCOS treatment (Cherlin et al., 2024). Pain prevalence in PCOS participants was initially reported at 30.1%, 25.8%, and 22.6% for abdominal and pelvic pain before their first prescription of COCPs, metformin, and spironolactone, respectively. After a minimum of 3 months of treatment, all groups demonstrated significant reductions in pain prevalence (Cherlin et al., 2024). COCPs reduce abdominal and pelvic pain prevalence by 5% and dysmenorrhea prevalence by 3.2% (Cherlin et al., 2024). Spironolactone, an anti-androgenic medication, reduces abdominal and pelvic pain prevalence by 7.5% and dysmenorrhea prevalence by 2.5% (Cherlin et al., 2024). It counteracts androgen-mediated inflammation, a major contributor to pain in PCOS, though its use is contraindicated during pregnancy due to risks like male fetal feminization (Patibandla et al., 2025; Armanini et al., 2016). Metformin provides modest pain relief, with a 2.5% reduction in abdominal and pelvic pain prevalence (Cherlin et al., 2024). For patients with more severe or refractory pain, nonsteroidal anti-inflammatory drugs (NSAIDs) like ibuprofen reduce prostaglandin-mediated inflammation by inhibiting cyclooxygenase-1 and cyclooxygenase-2, key enzymes in producing PGE2 (Banaszewska et al., 2022). Elevated PGE2 levels in PCOS contribute to inflammation and ovarian androgen production. By lowering PGE2, ibuprofen not only alleviates pain but also decreases androgen synthesis (Banaszewska et al., 2022). It is found in clinical studies that a 3-week course of ibuprofen reduces total testosterone by 21% and free testosterone by 28%, offering benefits for pain linked to hyperandrogenism (Banaszewska et al., 2022). However, due to risks such as gastric ulcer formation and other gastrointestinal side effects, long-term use of ibuprofen is not recommended (Sohail et al., 2023). When other therapies are ineffective, neuromodulators such as gabapentin or pregabalin are sometimes used for long-term pelvic pain, although their clinical efficacy remains uncertain (Lewis et al., 2016). These drugs modulate pain signaling pathways by binding to the α2δ subunit of voltage-gated calcium channels, reducing neuronal excitability and alleviating pain (Patel et al., 2016; Field et al., 2006). However, large trials in chronic pelvic pain have shown limited benefit (Horne et al., 2020), and these agents have not yet been evaluated in PCOS-related pain, where distinct neuroendocrine and inflammatory mechanisms may underlie nociceptive processing.

Lifestyle modification, which includes dietary, behavioral, or exercise changes, is first line of treatment for PCOS (Dennett et al., 2015). It has been demonstrated that behavioral therapies like stress management, cognitive-behavioral therapy, and mindfulness-based techniques can reduce the anxiety and depressive symptoms frequently linked to PCOS, while also indirectly improving adherence to other treatments (Almhmoud et al., 2024). Regular physical activity and exercise-based programs exert beneficial effects on metabolic function and systemic inflammation. For instance, following a 12-week high-intensity exercise program, insulin sensitivity was improved by 16%, suggesting reduced inflammatory tone and enhanced metabolic health (Patten et al., 2020). Progressive resistance training has been shown to improve muscle strength and decrease depression and anxiety scores in women with PCOS, highlighting its physical and psychological benefits (Almhmoud et al., 2024). Yoga therapy has also become a valuable lifestyle tool, as it has been found to regulate menstruation, enhance ovulation and ease pelvic pain by promoting pelvic blood circulation, as well as to reduce stress (Shrivastava et al., 2022). Dietary changes, such as adopting low-glycemic index or Mediterranean-style diets, complement exercise by reducing systemic inflammation and insulin resistance (Scannell et al., 2022). Studies have also shown that a high-fiber, complex carbohydrate, monounsaturated fat, Mediterranean-style diet improves body composition and decreases inflammatory markers such as adiponectin, IL-6, and CR (Schwingshackl and Hoffmann, 2023; Barrea et al., 2019). Collectively, these anti-inflammatory and metabolic benefits may help modulate pain perception and improve overall quality of life in PCOS; however, it should be noted that most lifestyle studies have not directly assessed pain outcomes, and their potential analgesic effects remain inferred rather than demonstrated.

The variability in pain expression among women with PCOS may also influence therapeutic outcomes. Although formal studies stratifying treatment response by pain status are lacking, existing evidence suggests that patients with more pronounced inflammatory or pain symptoms may respond differently to standard interventions, such as combined oral contraceptives, metformin, or anti-androgens, which improve hormonal balance and metabolic parameters and may indirectly alleviate pain by reducing systemic inflammation (Oguz and Yildiz, 2021; Dokras, 2016; Georgia, 2024; Dunaif, 2008). However, individuals exhibiting chronic or centrally sensitized pain often require multimodal management strategies, including targeted anti-inflammatory, neuromodulatory, or physiotherapeutic interventions, to achieve optimal outcomes (Sacca et al., 2024). Future research should therefore evaluate whether pain phenotype serves as a clinical predictor of treatment response in PCOS.

## 8 Emerging therapies in PCOS-related pain

To gain deeper insight into PCOS pathogenesis and the related pain sensitivity, ongoing studies are moving beyond traditional approaches to explore new molecular pathways. This research is focused on uncovering potential targets for reducing the inflammation, metabolic dysfunction, and pelvic pain seen in PCOS. Particularly involved is the endocannabinoid system (ECS), a central and peripheral neuromodulatory system defined by the cannabinoid 1 receptor (CB_1_R) and the cannabinoid 2 receptor (CB_2_R) which are both G-protein coupled receptors (Maia et al., 2017). CB_1_R are largely expressed in the brain, while CB_2_R are primarily expressed in peripheral immune cells, but are also found in hippocampal neurons and glial cells of the central nervous system (Bie et al., 2018). Both receptors can be activated by endogenous lipid ligands including N-arachidonoylethanolamine (anandamide; AEA) and 2-arachidonoylglycerol (2-AG) (Walker et al., 2019). ECS signaling, modulated by the enzymes fatty acid amide hydrolase (FAAH) and monoacylglyceride lipase (MAGL), is important in maintaining hormonal balance, inflammation, and energy homeostasis (Walker et al., 2019). However, evidence linking ECS dysregulation to PCOS is largely preclinical or correlative, and causality has not been established (Przybycień et al., 2022). As the condition is associated with obesity and hyperinsulinemia, factors that contribute to type II diabetes, it is possible that the ECS influences progression of disease via its control of these metabolic processes (Walker et al., 2019). Specifically, AEA, through activation of CB_1_R on pancreatic islet cells, has been shown to stimulate insulin secretion and promote insulin resistance (Cui et al., 2017a), both of which are common features of PCOS. Increased adiposity, which frequently coexists with PCOS, is associated with AEA and 2-AG dysregulation, further exacerbating insulin resistance (Walker et al., 2019). This metabolic imbalance, coupled with hyperinsulinemia’s effect on ovarian steroid hormone production by blocking LH, contributes to the cessation of follicular development, infertility, and anovulation (Motta, 2012). Endometrial FAAH was found to be reduced in women with PCOS compared to controls, suggesting possibly elevated AEA levels that can potentiate disease progression (Cui et al., 2017a).

Given its roles in metabolic and inflammatory regulation, the ECS has been proposed as a potential pharmacologic target in PCOS. Experimental work with FAAH inhibition suggests possible antinociceptive and anti-inflammatory actions via increased endocannabinoid tone; nevertheless, no clinical trials have evaluated ECS-directed therapies in PCOS, and safety and efficacy remain unknown (Walker et al., 2019; Przybycień et al., 2022; Zanfirescu et al., 2022; Santoso and De Ridder, 2023). FAAH inhibitors may exert their activity by elevating the levels of endocannabinoids such as AEA, modulating the endocannabinoid tone and potentially having antinociceptive and anti-inflammatory effects that can be applied to PCOS-related pain and inflammation (Zanfirescu et al., 2022; Ahn et al., 2009). Limited clinical observations indirectly support ECS modulation, for example, the parallel administration of the antiandrogenic hormonal contraceptive Diane-35 with metformin in women with PCOS was associated with normalization of circulating AEA levels (Cui et al., 2017b; Wooltorton, 2003). Moreover, the intricate interplay between the ECS and steroid hormones, notably estrogen, also gives it significance in the medical field. It has been found that estradiol benzoate, for example, causes a change in the expression of ECS components (CB_1_R, CB_2_R, FAAH) in the rat uterus, in an estrogen receptor-dependent way, indicating potential for hormonal regulation of the ECS in reproductive tissues (Maia et al., 2017). Given concerns about reproductive effects of cannabinoid exposure in young females, any therapeutic application would require rigorous evaluation of benefit–risk in the target population (Najafi et al., 2025).

The TRPV1 nociceptive ion channel is responsible not only for thermal and chemical nociception but also for inflammatory and neuropathic mechanical hyperalgesia, contributing to heightened pain sensitivity and neuroinflammation (Ma and Quirion, 2007; Gao et al., 2024; Holzer, 2008). In male C57BL/6 mice, diosgenin, a natural steroidal saponin, inhibited mechanical and thermal hypersensitivity by antagonizing TRPV1 and suppressing inflammation in DRG, without altering body temperature (Rahman et al., 2022). Notably, despite the induction of pain following acute TRPV1 activation by agonists such as capsaicin, prolonged/repeated stimulation has been shown to lead to receptor desensitization with analgesic effects (Gao et al., 2024). Collectively, these data support TRPV1 as a potential target for the treatment of visceral and inflammatory pain in PCOS.

Peroxisome proliferator-activated receptor (PPAR) signaling is being explored for its potential use in future PCOS therapeutics. PPARs are a family of nuclear receptor proteins that act as ligand-activated transcription factors. PPARs have three isoforms that regulate gene expression for lipid and carbohydrate metabolism, energy homeostasis, and inflammation (Wang, 2010; Contreras et al., 2013). Most relevant to PCOS, PPAR-γ has a key role in adipocyte differentiation, insulin sensitivity, and lipid metabolism, and its dysregulation has been directly implicated in PCOS pathogenesis (Wang et al., 2014). DHEA-induced PCOS in sexually immature female Sprague-Dawley rats was linked with a significantly reduced expression of PPAR-γ in adipose tissue at the mRNA and protein levels, suggesting that lipid metabolism disturbances via PPAR-γ suppression may contribute to hyperandrogenemia and metabolic dysfunction in PCOS (Wang et al., 2014). In addition, PPAR-γ also modulates granulosa cell proliferation and steroidogenesis, and its dysregulation may impair follicular development, contributing to ovarian dysfunction in PCOS (Zhao et al., 2025). Therefore, the administration of synthetic PPAR-γ agonists, so called thiazolidinediones, which are widely used to improve insulin resistance in type 2 diabetes (Quinn et al., 2008), has also shown therapeutic efficacy in PCOS management. In addition to improving peripheral insulin sensitivity, these agents also appear to have direct effects in the ovary, regulating steroid secretion, restoring ovulation, and increasing pregnancy rates (Froment and Touraine, 2006). This identifies PPAR-γ as a promising research focus for therapeutic modulation of both the metabolic and reproductive abnormalities seen in PCOS.

Research has also focused on endocannabinoid-like lipid mediators, including palmitoylethanolamide (PEA) and oleoylethanolamide (OEA) (Clayton et al., 2021). These compounds function as endogenous activators of PPAR-α and, to a lesser extent, PPAR-γ, distinct from their lack of direct binding to CB_1_R or CB_2_R (Lo et al., 2005; Guzmán et al., 2004). Preclinical models show that PEA provides analgesic and anti-inflammatory effects by suppressing mast cell activity and immune cell infiltration through PPAR-α–dependent pathways (Keppel Hesselink et al., 2013; Di Cesare Mannelli et al., 2013). For instance, in male C57BL/6 mice, PEA treatment significantly ameliorated mechanical hyperalgesia and inflammation in a chronic constriction injury model and was also shown to be neuroprotective, preserving nerve structure, all absent in PPAR-α knockout mice, confirming the PPAR-α signal-dependent analgesic and neuroprotective actions of PEA (Di Cesare Mannelli et al., 2013). OEA works to reduce appetite and to enhance lipolysis while improving insulin sensitivity, which benefits PCOS patients experiencing metabolic problems (Pouryousefi et al., 2022; Yang et al., 2007). In a recent clinical trial, daily supplementation with OEA significantly improved insulin sensitivity and decreased inflammatory markers (CRP, TNF-α) in PCOS women, further supporting its role in correcting metabolic dysfunction in PCOS (Shivyari et al., 2024). The interaction of PEA and OEA with PPARs instead of cannabinoid receptors generates a distinctive dual-modulatory impact, which may address both pain symptoms and metabolic dysfunctions. Limited clinical evidence exists for PCOS, but mechanistic foundations justify further investigation into these compounds for non-hormonal and anti-inflammatory metabolic therapy in patients suffering from obesity and chronic pelvic pain. Overall, ECS-, TRPV1-, and PPAR-based approaches remain hypothesis-generating, and their translational relevance and safety in PCOS require confirmation in well-designed clinical studies (Walker et al., 2019; Przybycień et al., 2022).

Repurposing existing anti-inflammatory and metabolic drugs may be a viable approach to treat pain and metabolic dysfunction in PCOS. In female C57BL/6 mice with DHEA (Dehydroepiandrosterone) and high-fat diet–induced PCOS, the Phosphodiesterase-4 inhibitor roflumilast improved ovarian function by increasing primary, preantral, and antral follicles, reducing cystic follicles and testosterone levels, as well as enhancing progesterone levels (Ji et al., 2023). The treatment also suppressed granulosa cell apoptosis and reduced inflammation by lowering TNF-α, IL-6, increasing the protein expression of the powerful anti-inflammatory cytokine, IL-10, as assessed by ELISA, as well as decreasing lipid accumulation in ovarian tissue (Ji et al., 2023). Similarly, glucagon-like peptide-1 receptor agonists, which were initially developed to treat type 2 diabetes, help improve insulin sensitivity and reduce appetite while supporting weight loss, and improving menstrual regularity and natural pregnancy rates for women diagnosed with PCOS (Han et al., 2019; Lamos et al., 2017). Clinical studies demonstrate that liraglutide and exenatide effectively reduce body weight in women with PCOS, either alone or when used with metformin (Lamos et al., 2017). In some trials, modest reductions in androgen levels and improvements in menstrual frequency were also observed (Lamos et al., 2017). These agents are not traditional analgesics but function as systemic metabolic modulators and could help patients suffering from both endocrine disorders and pain symptoms. They can also reduce low-grade inflammation and improve insulin sensitivity, which may in turn reduce pain symptoms in patients who are both obese and have chronic inflammatory disorders (He et al., 2023; Mishra et al., 2024).

Specialized pro-resolving lipid mediators (SPMs), including resolvins, protectins, maresins, and lipoxins, are endogenous lipids derived from omega-3 fatty acids like eicosapentaenoic acid and docosahexaenoic acid, orchestrating the resolution of inflammation (Julliard et al., 2022; Basil and Levy, 2016). SPMs are potent endogenous anti-hyperalgesic agents in several pain-associated disease paradigms, and they modulate pain perception through TRP channels (as their first endogenous inhibitors) and opioid receptors (Leuti et al., 2021). D-series resolvins, tested in male CD1 mice, were shown to attenuate mechanical allodynia in chemotherapy-induced peripheral neuropathy and formalin-induced inflammatory pain models (Luo et al., 2019b). In Sprague-Dawley rats, intrathecal administration of resolvins around the time of thoracotomy surgery effectively prevented the chronic post-operative hyperalgesia (Wang and Strichartz, 2017). Maresin 1, studied in C57BL/6 mice, suppressed TRPV1 activity in trigeminal nociceptive neurons and synaptic plasticity in models of temporomandibular joint inflammation (Park, 2015). Additionally, Protectin D1 attenuated neuropathic pain in CD1 mice following nerve injury by inhibiting spinal glial activation and proinflammatory responses, protecting dorsal root ganglion neuron function, and spinal cord synaptic plasticity (Xu et al., 2013). Collectively, these findings support the role of SPMs as a novel class of therapeutics for treating chronic inflammatory and neuropathic pain.

The kynurenine pathway (KP) is another prospective therapeutic target for managing PCOS, particularly due to its role in inflammation (Sacca et al., 2024; Jovanovic et al., 2022). The KP is the metabolic pathway of tryptophan that produces serotonin and other metabolites linked to mood disorders, neurodegenerative diseases, and chronic pain conditions, including PCOS (Sacca et al., 2024). Inflammation is one explanation for the link between PCOS and KP. Expanding on this, progesterone specifically has been found to inhibit the proinflammatory indoleamine 2,3-dioxygenase pathway of the KP. The fact that oral contraceptive pills usually contain progesterone means they also may work to relieve PCOS pain in part through this same action (Sacca et al., 2024). Therefore, KP’s manipulation is a novel way to approach PCOS pain.

## 9 Limitations and future directions

Future research directions should highlight multi-target strategies that involve ligands that influence both the endocannabinoid system and PPAR pathways, as well as agents that merge metabolic correction with central or peripheral pain modulation (Przybycień et al., 2022). These agents may provide a distinctive solution to PCOS pathology by linking metabolic dysfunction with nociceptive sensitization. Additional considerations should embrace the importance of individualized treatment approaches as they enable patients with obesity-related or inflammation-driven PCOS phenotypes to receive precise interventions tailored to their metabolic and hormonal needs (Teede et al., 2018). Effective management of PCOS pain needs new therapeutic development and essential modifications to clinical trials and practice to address pain as a primary symptom. Future studies are needed to assess new therapeutic treatments thoroughly and should include standardized pain rating scales and patient-reported outcomes as essential components. Additional mechanistic research is required to establish whether the pathophysiology of PCOS plays a direct role in both the emergence and ongoing presence of pain (Sacca et al., 2024). Indeed, it may be that the underlying pathophysiology related to PCOS may serve as a primer or ‘hit’ in the development in pathological pain. That is, small but physiologically relevant changes in PCOS-related hormone levels, even in patients that report no observable pain pre-injury, may be sufficient to induce post-injury or post-surgical pathological pain. These avenues of additional research may enable the conversion of agents initially intended for metabolic, hormonal and immune pathways into tools for phenotype-specific pain management plans.

Despite these promising directions, several limitations should be acknowledged when interpreting the current conclusions. Most evidence linking PCOS with chronic pain is derived from cross-sectional or observational data, limiting causal interpretation and introducing possible recruitment bias toward symptomatic cohorts. Pain outcomes are often self-reported and lack standardized quantitative assessments, which may underestimate the burden and complexity of pain. Furthermore, studies rarely differentiate among PCOS phenotypes, such as hyperandrogenic, metabolic-dominant, or normoandrogenic subtypes, despite their distinct hormonal and inflammatory profiles. Finally, there is a paucity of interventional trials evaluating whether therapeutic responses differ based on pain severity or phenotype, underscoring the need for well-designed longitudinal and mechanistic investigations to establish causality and inform personalized pain management strategies.
